# Targeted Chromatin Capture (T2C): a novel high resolution high throughput method to detect genomic interactions and regulatory elements

**DOI:** 10.1186/1756-8935-7-10

**Published:** 2014-06-16

**Authors:** Petros Kolovos, Harmen JG van de Werken, Nick Kepper, Jessica Zuin, Rutger WW Brouwer, Christel EM Kockx, Kerstin S Wendt, Wilfred FJ van IJcken, Frank Grosveld, Tobias A Knoch

**Affiliations:** 1 Department of Cell Biology, Erasmus MC, Dr. Molewaterplein 50, 3015GE Rotterdam, The Netherlands; 2 Deutsches Krebsforschungszentrum (DKFZ) & BioQuant, Im Neuenheimer Feld 280, Heidelberg 69120, Germany; 3 Center for Biomics, Erasmus MC, Dr. Molewaterplein 50, 3015GE Rotterdam, The Netherlands

**Keywords:** Chromatin conformation capture, Long range interactions, Enhancers, Promoters

## Abstract

**Background:**

Significant efforts have recently been put into the investigation of the spatial organization and the chromatin-interaction networks of genomes. Chromosome conformation capture (3C) technology and its derivatives are important tools used in this effort. However, many of these have limitations, such as being limited to one viewpoint, expensive with moderate to low resolution, and/or requiring a large sequencing effort. Techniques like Hi-C provide a genome-wide analysis. However, it requires massive sequencing effort with considerable costs. Here we describe a new technique termed Targeted Chromatin Capture (T2C), to interrogate large selected regions of the genome. T2C provides an unbiased view of the spatial organization of selected loci at superior resolution (single restriction fragment resolution, from 2 to 6 kbp) at much lower costs than Hi-C due to the lower sequencing effort.

**Results:**

We applied T2C on well-known model regions, the mouse *β-globin* locus and the human *H19/IGF2* locus. In both cases we identified all known chromatin interactions. Furthermore, we compared the human *H19/IGF2* locus data obtained from different chromatin conformation capturing methods with T2C data. We observed the same compartmentalization of the locus, but at a much higher resolution (single restriction fragments *vs.* the common 40 kbp bins) and higher coverage. Moreover, we compared the *β-globin* locus in two different biological samples (mouse primary erythroid cells and mouse fetal brain), where it is either actively transcribed or not, to identify possible transcriptional dependent interactions. We identified the known interactions in the *β-globin* locus and the same topological domains in both mouse primary erythroid cells and in mouse fetal brain with the latter having fewer interactions probably due to the inactivity of the locus. Furthermore, we show that interactions due to the important chromatin proteins, Ldb1 and Ctcf, in both tissues can be analyzed easily to reveal their role on transcriptional interactions and genome folding.

**Conclusions:**

T2C is an efficient, easy, and affordable with high (restriction fragment) resolution tool to address both genome compartmentalization and chromatin-interaction networks for specific genomic regions at high resolution for both clinical and non-clinical research.

## Background

A number of recent studies have shown that the genome is organized in self-associating domains [1] that are separated by linker regions. These so-called ‘topological domains’ or ‘topological associated domains’ generally range from 300 kilobasepairs (kbp) to 1 megabasepairs (1 Mb) and consist of a series of different types of chromatin loops in agreement with earlier models of the genome ([2] and references therein).

One loop is defined as two distant chromatin regions coming, spatially, into close proximity (interact with each other), thereby creating DNA loops. Such ‘long-range interactions’ have been first observed between promoters and distant enhancers ([3,4] and references therein) and can bring DNA elements together that are separated by a large distance on the linear DNA strand ([5,6] and references therein). These regulatory elements (enhancers or silencers) are short sequences containing several binding sites for transcription factors, which regulate the activation (reviewed in [7]) repression (reviewed in [8]) genes and their subsequent transcription (reviewed in [9]). In the linear genome the distance between enhancer(s) and gene can be quite large, for example, the sonic hedgehog (shh) enhancer is located about 1 Mb away from its target gene *Shh*[10]. Changes or differences within these elements and their interaction with genes can be responsible for changes in gene expression [11], causing intrinsic differences between individuals, disease susceptibility, and disease progression.

A number of chromatin loops are thought to be purely structural, that is, to enable the folding of the genome creating distinct topological domains, while other loops have a function in the expression of genes. Loops of the latter type are frequently found within topological domains, but are less frequently observed between different topological domains [1,12]. These regulatory chromatin loops change and depend on a large number of proteins including Ctcf [13], cohesin [14], and a series of transcription factors [15-18], which are mostly involved in the transcriptional regulation of genes within the domain.

The recent refinements of the genome structure were largely due to the chromosome conformation capture (3C) technique which allowed the rapid identification of chromatin regions residing in close proximity [19,20]. The basic principle of the 3C technique is that segments, which are spatially in close proximity within the cell nucleus, can be tethered together by cross-linking. After cross-linking and restriction enzyme digestion of the genome, the proximal segments remain covalently linked and segment ends can be, subsequently, ligated in dilute conditions. The ligation products can be analyzed using PCR-based methods [19]. A number of different 3C-type techniques have been developed to answer different biological questions including: 3C/3C-qPCR [19,21,22], 3C-seq/4C-seq [23,24], 4C (3C-on-a chip) [25-27], Chromatin Interaction Analysis by Paired-End Tag Sequencing (ChIA-PET) [28], 5C (3C carbon copy) [29], and Hi-C [30]. All these techniques have their own advantages and limitations (Table 1) and have provided very valuable information on chromosomal interactions and gene transcription mechanisms [20,25,30,31]. 3C and 4C are quite work- and cost-intensive, given that they are only one-to-one fragment and one-to-all fragment techniques, respectively. Prior knowledge of the locus is necessary to define the region of interest.

**Table 1:** **Comparison between different chromatin conformation capturing techniques (adopted and modified from **[23]**)**

**Method**	**Applications**	**Advantages**	**Limitations**
**3C-qPCR**	One-to-one	Simple analysis	Laborious, requires knowledge of the locus and proper controls
**3C-seq/4C-seq**	One-to-all	Good resolution, good signal-to-noise ratio	Restricted to single viewpoint per experiment when multiplexing several viewpoints, analysis requires extra bioinformatics expertise, not an all-to-all genome-wide method
**3C-on-chip (4C)**	One-to-all	Relatively simple data analysis	Poor signal-to-noise ratio, difficult to obtain genome-wide coverage
**5C**	Many-to-many	Identifies interactions between many individual fragments	Very laborious, no genome-wide coverage, primer design can be challenging. Analysis requires advanced bioinformatics expertise
**Hi-C**	All-to-all	Explores the genome-wide interactions between all individual fragments	Very expensive, requires a large sequence effort to obtain sufficient coverage, approximately 10 to 40 kbp resolution, requires advanced bioinformatics expertise
**T2C**	Many-to-all	Explores the interactome of a selected region in cis but also in trans, high (restriction fragment) resolution, cheaper than Hi-C and 5C, requiring only half a lane of Illumina HiSeq2000	Is restricted to the selected regions of the genome, requires advanced bioinformatics expertise

The analysis of the interactions of several viewpoints with the aforementioned techniques in 3C and 4C is possible, but the choice for several viewpoints will increase the costs and work effort linearly. However, the number of viewpoints can also be limited due to the (often) limiting amount of available cell material. 5C is demanding in primer design and allows the analysis of interactions only among the primer designed fragments. Furthermore, genome-wide coverage is not possible. Hi-C is very expensive as it requires extremely deep sequencing in order to cover the whole genome, even at a relatively low resolution of 40 kbp. The most recent Hi-C data analysis has used a new algorithm and provided a genome-wide interaction map of 10 kbp resolution. However, an enormous amount of sequencing is required (3.4 billion mapped paired-end reads from six biological replicates) [32]. Such effort is not affordable for most research groups and, in addition, the scientific interest is most of the time focused on a specific question involving a limited set of specific loci or domains. Hence, there is a need for a technique which eliminates most of the aforementioned limitations.

Here we present Targeted Chromatin Capture (T2C), a new 3C method, which does not involve a massive sequencing effort, but which results in a high resolution map of interactions for particular loci of interest. We used the well-studied human *H19/IGF2* locus and compared the results of our new method with data from other chromatin conformation capturing techniques. Using the mouse *β-globin* locus we demonstrated that the method can reliably identify chromatin structural changes between different tissues and also allows the study of the role of individual transcription factors in the chromatin architecture.

### Overview of the procedure

To overcome the aforementioned problems of the 5C and Hi-C techniques we have developed the novel method T2C. The method has the advantage that it allows the analysis of the structure of the genome and all the interactions of selected regions of the genome at high resolution (single restriction fragments) without a massive sequencing effort and associated costs.

T2C employs a selective enrichment of the 3C ligation products in preselected regions of interest in order to identify their interactions within a domain as well as the compartmentalization of one or several specific regions of the genome. These regions can be continuous Mb sized genomic regions, but could also be a collection of smaller regions (a few kbp each). Every captured restriction fragment can be used as a single ‘4C-seq viewpoint’ and analyzed accordingly. The results of T2C provide a local interaction map at a restriction fragment-level resolution accompanied with a lower sequencing effort and less intricate bioinformatics analysis than Hi-C. T2C also overcomes the limits of 5C since it identifies not only interactions within the targeted region(s), but also interactions between the targeted region(s) and with regions outside of them.

In brief, we have designed sets of unique oligonucleotide probes (ranging from 62 to 90 nucleotides) specific for all the restriction fragments and as close as possible to the end of the first restriction site (*Mm* - HindIII + NlaIII digest, *Hs* - BglII + NlaIII digest) in our regions of interest, the mouse *β-globin* locus and the human *H19/IGF2* locus (see Methods). Alternative to continuous regions, separate genomic regions within one (or more) chromosomes could be analyzed simultaneously. The oligonucleotides are spotted on an array or can alternatively be captured on beads. Some fragment ends cannot be captured by a designed oligonucleotide due to the presence of repeat elements or the insufficient size of the restriction fragment end. Repetitive sequences are a general problem in all 3C-based methods, including Hi-C. The size limitation of the fragment end can be circumvented if necessary by a backup procedure with different enzymes (changing either the first or the second restriction enzyme or both), which generates a new set of end fragments or by mechanically shearing of the chromatin (instead of the second restriction enzyme digestion) which can result in fragment sizes of different length (see Discussion).

**Figure 1:**
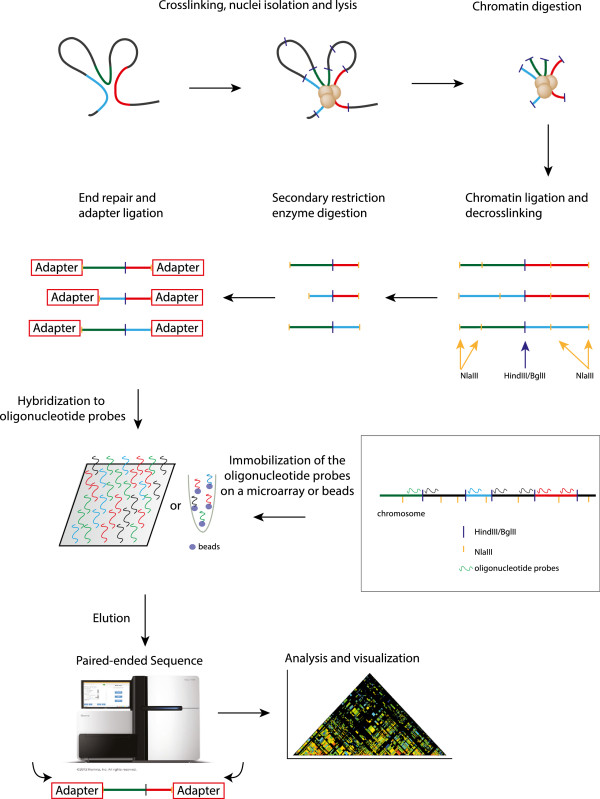
**Overview of the targeted chromosome capture (T2C) procedure.** Isolated cross-linked chromatin is digested with a restriction enzyme (dark blue lines) and ligated under diluted conditions to favor ligations between restriction fragments that are spatially in proximity. After de-cross-linking and a secondary digestion (orange lines), the overhangs are repaired followed by adapter ligation. Different address sequences can be used in the adapters for different samples to allow multiplexing of different samples (hybridization of different samples to the same set of oligonucleotides). The resulting library is hybridized to a set of unique oligonucleotides on an array or oligonucleotides in solution that are captured on beads. The unique oligonucleotides (green, red, black, and blue lines) are located as close as possible to the first restriction site. The hybridized DNA, which contains the library of all interactions from the selected area of the genome, is eluted and is pair-end sequenced on an Illumina HiSeq2000 followed by bioinformatic analysis and visualization of the chromatin interactions (that is, sequences in close proximity). Each point in the chromatin interaction map, represents an interaction (in restriction fragment resolution, each block represents the size of the restriction fragment) between two fragments in the genome.

The first steps of the preparation of the chromatin conformation capturing library are carried out as in 3C-seq [23]. Basically, chromatin is cross-linked, followed by digestion with a 6 bp recognition restriction endonuclease, ligation in diluted conditions and decross-linking of the DNA. The library is subsequently digested with a frequently-cutting 4 bp recognition restriction endonuclease or mechanically sheared to obtain small fragments containing the ligation site, followed by end-repair and ligation of an adapter. Within the adapter, different barcodes can be included that would allow multiplexing of different samples. The resulting library is hybridized to the specific oligonucleotide probe set representing the area(s) of interest (either on an array or in a bead capturing procedure) to enrich specifically for the interacting fragments of the region of interest (all fragments positioned at the ends of the original 6 bp-cut fragments after the second 4 bp-cut and eliminate all fragments internal to the 6 bp generated fragments). After extensive washing all ligation products including regions covered by the targeting-array are eluted and their sequence determined by Illumina-sequencing (Figure 1). The capture efficiency (the proportion of paired reads of total reads when at least one read of the paired end reads is located on a fragment represented by an oligonucleotide) is between 47% and 86% depending the cell type and the region (see Table 2).

**Table 2:** Summary of information about the different experiments

**Type**	**Genome assembly version**	**Coordinates oligo-nucleotide positions**	**Size of area of interest (Mb)**	**Median resolution (kbp)**	**Raw paired reads (n)**	**Paired reads that could be mapped to the whole genome (n)**	**Mapped paired reads between the region of interest and the whole genome (n)**	**Uniquely mapped paired-reads in the whole genome without self-ligation and and non-digestion (n)**	**Uniquely mapped paired-reads between the region of interest and the whole genome without self-ligation and non-digestion (n)**	**Uniquely mapped paired-reads inside the region of interest without self-ligation and non-digestion (n)**	**‘Interactions’ inside the region of interest (n)**	**Average number of reads/interaction in the region of interest (n)**
Mouse fetal liver	mm9	chr7: 109876329-111966581	2.1	2	65,165,916	9,300,108	5,716,401	4,559,952	2,723,515	557,763	4,057	137
Mouse fetal brain	mm9	chr7: 109876329-111966581	2.1	2	84,977,143	6,380,256	3,191,360	3,018,169	1,414,128	271,177	2,369	114
HB2	hg18	chr11: 1100646 - 3173091	2.1	4.1	51,952,969	13,813,662	12,127,051	5,503,770	4,745,779	1,929,245	8,989	215

Columns from left to right: Tissue type or cells; genome assembly version; summary of the positions of oligonucleotides (region of interest); the size and the median resolution of the area under investigation; the number of the raw paired-reads (before alignment, that is, all reads from the sequenator); the number of mapped paired reads that could be mapped back to the whole genome; the number of paired reads between the region of interest (fragments with oligonucleotides) and the whole genome; the number of uniquely mapped paired-reads in the whole genome after removal of the self-ligation and non-digestion events (See Methods); the number of uniquely mapped paired-reads between the region of interest (fragments with oligonucleotides) and the whole genome after removal of the self-ligation and non-digestion events; the number of uniquely mapped paired-reads inside the region of interest after removal of the self-ligation and non-digestion events; the number of ‘interactions’ between fragments in the region of interest; average number reads per interaction. The capture efficiency and purification (enrichment) by hybridization is high (that is, how many reads from the region of interest (‘specific’ reads) are found when compared to total reads, that is, the reads that are from other areas of the genome and not containing a sequence from the area of interest (‘non-specific’ reads)). We find that the ‘specific’ reads represent 61%, 50%, and 88% of total reads (‘specific’ plus ‘non-specific’) for mouse primary erythroid cells, mouse fetal brain cells, and HB2, respectively, including the self-ligation and non-digestion events. By removing those events those numbers change to 60%, 47%, and 86%, respectively. This means for example that 60% of the fetal liver reads (2,723,515) represent 2.1 × 10^6^ bases (the region of interest) while the remaining 40% of reads represents 3.10^9^ bases (the whole genome), numbers that indicate a high level of enrichment by the hybridization step.

## Results

### T2C identifies known long-range interactions

**Figure 2:**
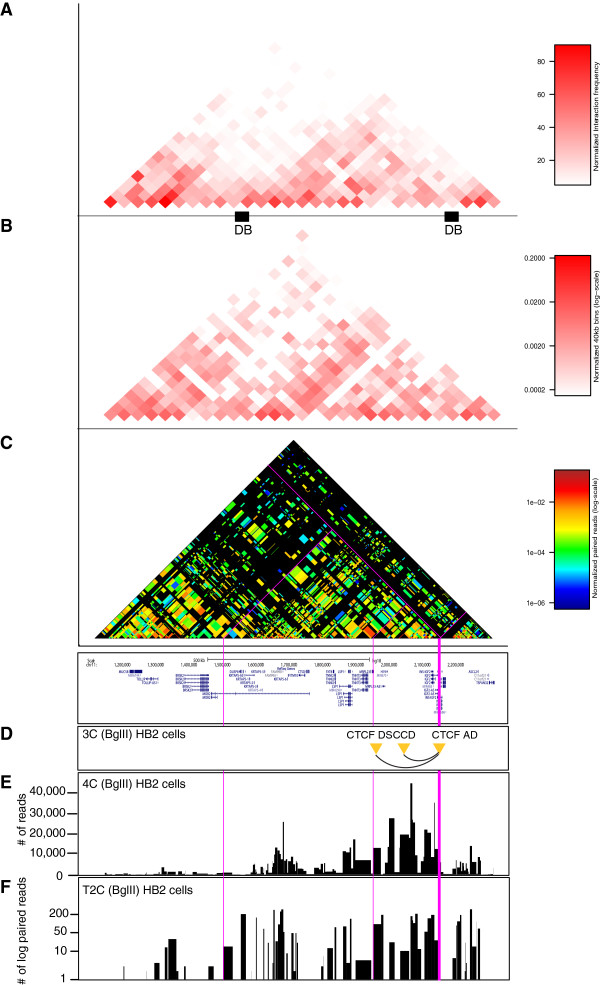
**Comparison of interactions detected by T2C for the human chr11p15.5 region with Hi-C and 4C-seq. (A)** Hi-C data generated by Dixon *et al.* for IMR90 cells covering the *H19/IGF2* region of interest, presented at a resolution 40 kbp with their respective domain boundaries (DB) depicted as black boxes [1]. **(B)** T2C interactions in HB2 cells at a 40 kbp resolution. The overall topological domain pattern observed by the two methods is similar (*r*_*s*_ = 0.64, *P* <2.2 × 10^-16^). **(C)** T2C interaction with their actual resolution at restriction fragment level. **(D)** Interactions detected by 3C [33]. The restriction fragments are indicated with yellow triangles. **(E)** 4C-seq interaction data [14], for a viewpoint close to the *IGF2* gene. **(F)** Interactions observed for a particular viewpoint by T2C plotted with logarithmic y-axis. The position of the viewpoint is indicated as bold pink line to allow a direct comparison between the methods. The thin pink lines indicate a couple of interaction fragments for ease of comparison.

We first have chosen the *H19/IGF2* region on human chromosome 11 to test and compare the method to other 3C methods. Previously, we analyzed the 3D-structure of the locus by 3C to study the role of cohesin and CTCF for chromosomal long-range interactions [33] and also generated 4C-seq data [14] (Figure 2). Hi-C interaction maps were retrieved for IMR90 cells [1].

We selected unique oligonucleotides mapping near the ends of 344 BglII generated fragments spanning 2.1 Mb around the *H19/IGF2* locus (Table 2). This set of 525 oligonucleotides was spotted on a capture array. A ligation fragment library was generated from the breast endothelial cell line 1-7HB2 (abbreviated HB2) after digestion with BglII and NlaIII according to the 3C-seq protocol [23] (see also Figure 1). The library was subsequently hybridized to the capture array. After elution from the capture array the captured DNA fragments were amplified by a PCR with low cycle number (12 cycles) and sequenced by paired-end Illumina sequencing (see Methods).

To demonstrate that T2C reveals a similar overall interaction pattern and compartmentalization of the locus as observed by Hi-C in IMR90 cells [1] we first binned the paired-reads into 40 kbp bins (Figure 2A, B). The interaction patterns at this level of resolution show that the topological domain is maintained between different cell types, HB2 [14]*versus* IRM90 [1] with a Spearman’s rank correlation coefficient *r*_*s*_ = 0.64 (*P* <2.2 × 10^-16^).

However, with T2C we obtained a chromatin interaction map at restriction fragment resolution (Figure 2C, each block represents one restriction fragment), revealing significantly more detail with respect to the general chromatin organization of the region when visualized by a logarithmic and rainbow-like colored interaction frequency. To first validate T2C in comparison to 3C and 4C-seq we compared the interactions of a single restriction fragment (CTCF AD viewpoint) [33] to interactions detected for this fragment by 3C [33] and 4C-seq [14] (Figure 2D, E, F). Although there are some variations in the read coverage of the individual interactions, similar interactions can be observed by both 4C-seq and T2C. Moreover, both methods detect interactions which we previously observed with 3C [33]. It should be noted that an important difference between 4C-seq and T2C is the number of PCR amplification cycles. For T2C this is on average 12 cycles (only after capture) whereas for 4C-seq it is 30 cycles. The lower number of cycles will give less PCR bias of the different fragments relative to each other, because fragments have different PCR efficiencies.

We conclude that the T2C method yields interaction data at a resolution identical to 4C-seq for the individual restriction fragments (median approximately 4 kbp resolution) and that when T2C is performed for a continuous region over 2 Mb it can reproduce the overall topological domain structure that was observed by Hi-C.

### T2C identifies different interaction networks based on different biological materials

**Figure 3:**
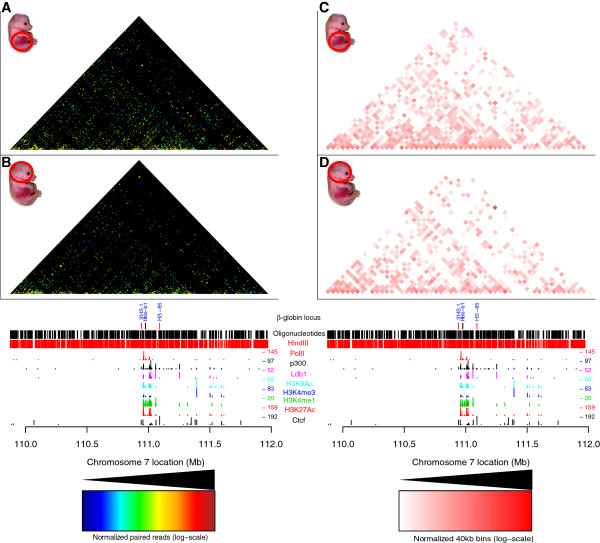
**Comparison of the compartmentalization and interactions for the *β-globin *locus.** T2C performed in a 2.1 Mb region around the *β-globin* locus for mouse primary erythroid cells **(A)** and mouse fetal brain cells **(B**) from E12.5 mice. The topological domain patterns between different biological materials are identical and are independent of the number of interactions. Analysis of the interactions obtained with T2C obtained from mouse primary erythroid cells **(C)** and mouse fetal brain cells **(D)** were plotted at 40 kbp resolution to compare T2C to the regular Hi-C binning. The overall topological domain pattern is similar in the two tissues. All the T2C interactions are normalized to the same color code (see color inset). The bottom tracks show a linear representation of the *β-globin* locus, the oligonucleotides probes positions (black lines), *Hin*dIII recognition sites (red lines) and the ChIP-seq derived binding sites of PolII (red lines), Ldb1 (purple lines) [38], Ctcf (black lines), p300 (black lines), and various histone modification markers (light blue, dark blue, green, and red) [37] in mouse erythroleukemia cells.

**Figure 4:**
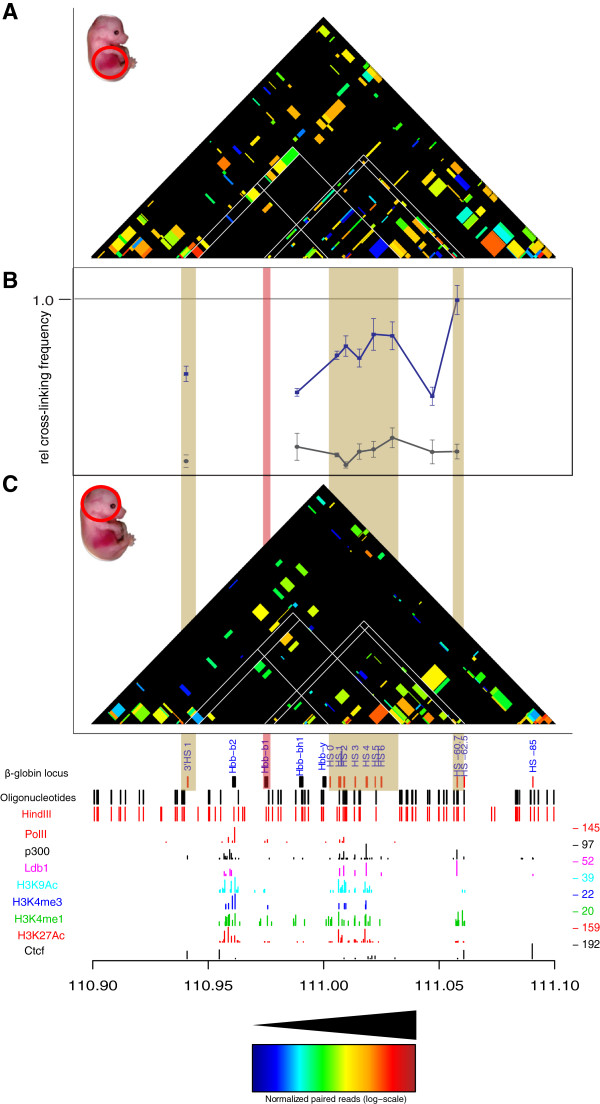
**Comparison of T2C with 3C-qPCR for the *β-globin *promoter.** T2C for mouse primary erythroid cells **(A)** and mouse fetal brain cells **(C)** from E12.5 mice, revealed the same interactions from the *β-globin* promoter when comparing them to 3C-qPCR **(B)**. The 3C-qPCR was adapted and modified from Drissen *et al.*[16] with blue line depicting the interactions for primary erythroid cells and with grey the interactions for mouse fetal brain cells from E12.5 mice. White lines indicate the areas of particular interest (such as 3’HS1, *β-globin* promoter, Locus Control Region (LCR) and 5′ HS-60/-62) in the *β-globin* locus. Interactions between LCR, the *β-globin* promoter and the 3′HS1 are lost in mouse brain cells. The shaded vertical bars indicate the comparison between the different panels. The red vertical bar indicates the *β-globin* promoter. All the T2C interactions are normalized to the same color code (see color inset). The bottom tracks show a linear representation of the *β-globin* locus, the oligonucleotides probes positions (black lines), *Hin*dIII recognition sites (red lines) and the ChIP-seq derived binding sites of PolII (red lines), Ldb1 (purple lines) [38], Ctcf (black lines), p300 (black lines), and various histone modification markers (light blue, dark blue, green, and red) [37] in mouse erythroleukemia cells.

**Figure 5:**
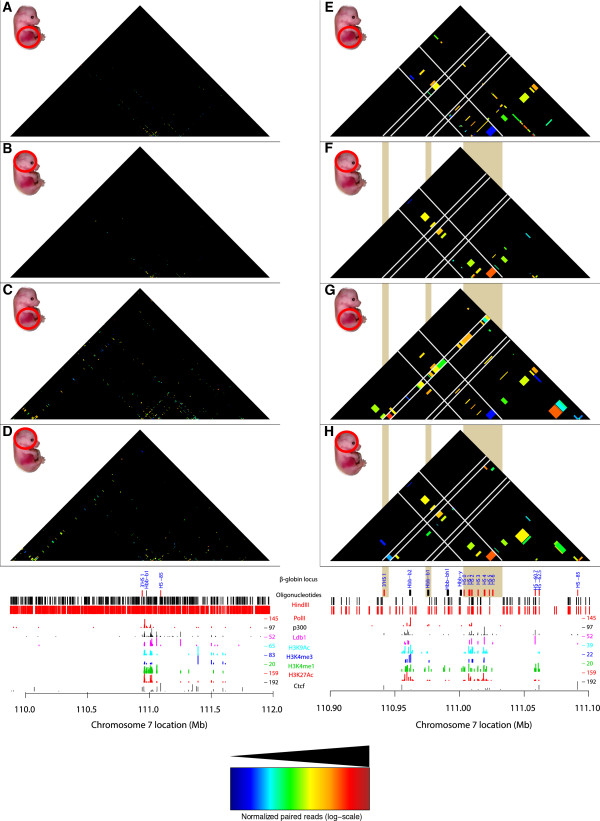
**T2C/ChIP-seq intersection plot.** A comparison of the interactions containing one or two fragments with a Ldb1 or Ctcf binding site. Interactions are plotted, at restriction fragment resolution, over a 2.1 Mb region around the *β-globin* locus for Ldb1 **(A, B)** or Ctcf **(C, D)** for mouse primary erythroid cells **(A, C)** and mouse fetal brain cells **(B, D)** from E12.5 mice. The topological sub-domain around the *β-globin* locus is clearly depicted in the mouse primary erythroid cells when compared to mouse brain cells. Focusing on the *β-globin* locus, T2C-intersection plots, at restriction fragment resolution, of interactions that contain a Ldb1 bound fragment **(E, F)** or a Ctcf bound fragment **(G, H)**, for mouse primary erythroid cells **(E, G)** and mouse brain cells **(F, H)**. White lines indicate particular areas of interest (like 3′HS1, the *β-globin* promoter and the Locus Control Region (LCR)) in the *β-globin* locus. The mouse primary erythroid cells interactions between LCR, *β-globin* promoter, and 3′HS1 are lost in mouse brain cells. The shaded vertical bars indicate the comparison between the different panels. All the interactions are normalized to the same color code (see color inset). The bottom tracks show a linear representation of the *β-globin* locus, the oligonucleotides probes positions (black lines), *Hin*dIII recognition sites (red lines) and the ChIP-seq derived binding sites of PolII (red lines), Ldb1 (purple lines) [38], Ctcf (black lines), p300 (black lines), and various histone modification markers (light blue, dark blue, green, and red) [37] in mouse erythroleukemia cells.

Next we used the extensively characterized mouse *β-globin* locus as a model system to show that the T2C method can detect reliably conformational changes due to activation of the genes *in vivo* at high resolution (Figures 3 and 4). We further showed, with an intersection between ChIP-seq derived chromatin protein data and T2C, that chromatin proteins may be involved in forming or maintaining the 3D structure of the genome (Figure 5).

The mouse *β-globin* locus undergoes structural changes upon activation in erythroid tissue [20,34,35], but is surrounded by silent olfactory receptor genes, which are only expressed in the olfactory epithelium. The major difference between the *H19/IGF2* locus and the *β-globin* locus is that the *β-globin* locus is embedded in a large area of inactive genes. Thus two patterns of interactions may be expected in erythroid cells, those important for the globin locus and those present in inactive chromatin. We selected a region of 2.1 Mb around the locus (Table 2) containing 719 restriction fragments of the restriction enzyme HindIII (6 bp recognition site). About 800 oligonucleotide probes were designed close to the ends of the fragments. To analyze the locus in its active state we used primary erythroid cells from fetal liver which were compared to fetal brain cells as a model of inactive loci. Based on results from previous 3C studies of the locus [20,35] we expected in primary erythroid cells a higher number of interactions around the *β-globin* gene and between the *β-globin* gene and its regulatory elements.

The analysis of the hybridized fragments shows that almost the entire 2.1 Mb appears to be part of one topological domain (with two possible subdomains, one of which contains the *β-globin* locus) with the next domain starting near the end of the selected sequences (due to the repetitive sequences and the borders of the region of interest, that topological domain cannot be depicted clearly, in agreement with Dixon *et al.*[1]) both in mouse primary erythroid cells (Figure 3A, right hand side) and mouse fetal brain cells (Figure 3B) with many interactions within the topological domain (Figure 3C and 3D). Although the topological domain structure between the different biological materials is similar, there appear to be fewer interactions in mouse fetal brain cells relative to mouse primary erythroid cells due to the inactivity of the locus in the brain (Figure 3). Focusing on the *β-globin* region, all the well-known interactions in the *β-globin* locus are detected in the primary erythroid cells. The known interactions, such as between the *β-globin* promoter and Locus Control Region (LCR) (Figure 4B, adapted and modified from Drissen *et al.*, with blue line depicting the interactions for primary erythroid cells and with grey the interactions for mouse fetal brain cells) and between the LCR-3′HS1 are clearly visualized [16,20,35] (Figure 4A). These interactions are absent from the fetal brain sample (Figure 4C). Furthermore, the main regulatory region (HS1-6) shows the well-known interaction with the *β-globin* genes and HS1 at the 3′end of the locus in fetal liver cells but not in brain [16,20]. In addition, for the *β-globin* promoter we identify a few additional interactions further away than the ones previously reported. These are located even approximately 1 Mb far from the *β-globin* promoter (Figure 3A). It is unknown whether these interactions are related to the functioning of the *β-globin* genes or whether these DNA elements are in close proximity due to the folding of the domain, although their absence in the fetal brain suggests they have a role in the regulation of the globin *β-globin*. In addition to the interactions *in cis*, the *β-globin* (*Hbb-b1*) gene and the LCR also contact a number of positions on other chromosomes.

### T2C in combination with ChIP-seq identifies factor specific interactions

**Figure 6:**
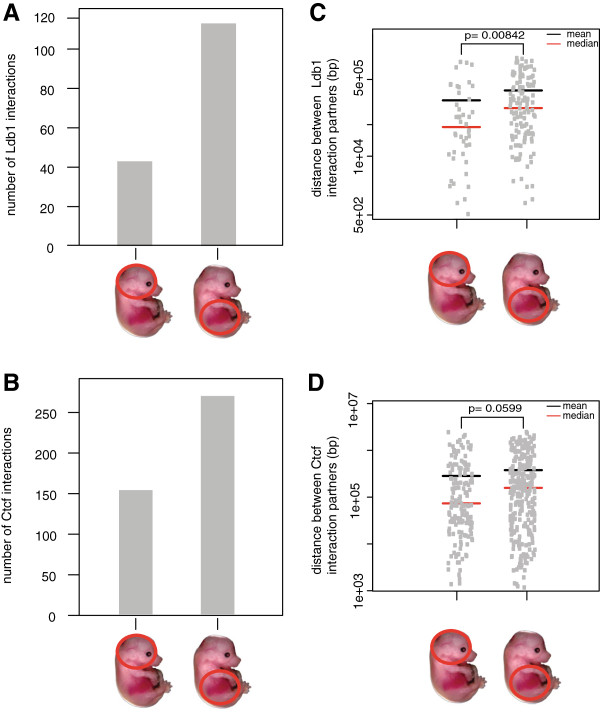
**The mean, median, and the number of T2C interactions for the Ldb1 or Ctcf containing fragments.** The number of Ldb1 **(A)** and Ctcf **(B)** interactions is lower in mouse fetal brain when compared to primary erythroid cells. Furthermore, the mean and the median of the distance between either Ldb1 **(C)** or Ctcf **(D)** interaction partners is lower in mouse fetal brain cells when compared to mouse primary erythroid cells. *P* values were calculated using the Mann–Whitney *U* test.

We also compared the interactions of the binding sites of an important regulatory transcription factor in mouse primary erythroid cells, the Ldb1 complex, and the insulator binding protein Ctcf (Figure 5A-D). Ldb1 is highly enriched on the β-globin locus and its LCR in mouse primary erythroid cells compared to fetal brain cells [36]. By visualizing only the restriction fragments containing the Ldb1 or Ctcf binding sites as determined by ChIP-seq in fetal liver derived mouse erythroleukemia cells (MEL) [37,38], we can immediately deduce in which interactions the Ldb1 complex (Figure 5E, F) or Ctcf (Figure 5G, H) are involved. In addition, we can identify the restriction fragments that represent gene promoter fragments (by Histone 3 Lysine 4 trimethylation (H3K4me3)) or enhancer fragments (marked by H3K4me1, that is, in the LCR, HS-60, and -62.5) or neither of these, by plotting the histone modifications ChIP-seq profiles [37]. Interestingly the 3′HS1 and HS-85 belong to the latter class and have robust Ctcf but not Ldb1 binding sites. This suggests that they are ‘structural’ elements which would fit with the observation that the deletion of the 3′HS1 results in a loss of looping but not in a decrease of *β-globin* mRNA levels [13]. In contrast the enhancer immediately 3′ of the *β-globin* enhancer is apparent, but it does not appear to interact with any distal elements. It is also clear that in mouse primary erythroid cells Ldb1 (Figure 6A) and Ctcf (Figure 6B) occupy restriction fragments that have more interactions with other positions in the locus when compared to mouse brain cells. In addition the median distance on the linear chromosome between two fragments in spatial proximity is larger in primary erythroid cells for both Ldb1 (Figure 6C) and Ctcf (Figure 6D) binding sites. This suggests that this area of the genome is less condensed. We conclude from these experiments that T2C indeed detects topological domains and the different interactions between and within domains. These interactions depend on the expression status of the genes such as the active *β-globin* locus in primary erythroid cells versus the same silent locus in fetal brain. In addition, the high level of resolution of the interactions allows novel observations such as shown for the *β-globin* locus Ldb1 and Ctcf binding sites and immediately shows which of these binding sites interact with each other and where they are positioned on the linear genome.

## Discussion

The importance of the role of chromatin interactions in the regulation of the gene transcription is well established [9,39-42]. However, there is still an increasing need for a quick, easy, and affordable technique to provide the information on chromatin interactions and the compartmentalization of the genome. T2C is affordable to most scientific groups and will meet in a satisfactory manner their needs for detecting high resolution chromatin organization of selected loci. Every restriction fragment can serve as a ‘viewpoint’ and all their interactions, either short or long or to other chromosomes (data not shown), can be identified. Thus multiple 3C-seq, 4C-seq or 5C experiments do not have to be performed. Moreover, with T2C the compartmentalization of the genome can be identified in the regions of interest without requiring the large sequencing effort of Hi-C, which would increase the costs tremendously. Furthermore, due to the T2C design, a better coverage and resolution of the locus is obtained when compared to other genome wide techniques (like Hi-C and 3C with its derivatives) using a 6 bp cutter as first restriction enzyme. Here we multiplexed two samples, but by multiplexing more than two samples the costs are likely to be reduced significantly without sacrificing the quality of the output. We have recently successfully used 13 samples per sequencing lane, including the *β-*globin locus which showed the same interactions (data not shown).

The resolution of T2C is based on the restriction enzyme used. Digesting cross-linked chromatin from primary erythroid cells and HB2 cells with HindIII or BglII, resulted in a median resolution of 2 kbp and 4.1 kbp, respectively (Table 2). That provides a significantly better resolution than the usual 40 kbp bins obtained with Hi-C. Furthermore, comparing T2C with 4C-seq [14] and Hi-C [1] for the *H19/IGF2* locus (Figure 2) and with already published 3C-qPCR data for the *β-globin* locus [16,20,35], the same topological domains and chromatin interaction networks were identified. Taken together, all these results reveal the strengths of the T2C as a tool to identify all the interactions and the compartmentalization of specific regions of the genome.

In addition, the T2C interactions are easily connected to the factors that play a role in these interactions or the type of elements (promoters/enhancers) involved in the interactions. Ldb1 and Ctcf are important proteins which mediate chromatin interactions. Ldb1 is an important transcription factor necessary for primitive mouse hematopoiesis and for the development of megakaryocytes [43,44] and controls essential hematopoietic pathways in mouse early development [45]. Depletion of Ldb1 is lethal for mouse embryos after E9.5 with severe effects such as impairment of hematopoietic and vascular development [46]. It is well established that the LCR has higher interaction frequencies with the *β-globin* locus in mouse primary erythroid cells comparing to mouse brain cells [16,20,35] and that Ldb1 is significantly enriched in the LCR region in mouse primary erythroid cells relative to mouse fetal brain cells [36] (Figure 5E *vs.* Figure 5F). Furthermore, Ctcf is an insulator binding protein known to be involved in chromatin conformation [33] and is enriched at the boundaries of topological domains [1]. Ctcf mediates long range interactions in the *β-globin* locus [13] (Figure 5C *vs.* Figure 5D and Figure 5G *vs.* Figure 5H). Hence, it is no surprise that for Ldb1 and Ctcf occupied restriction fragments we observe a higher number of interacting fragments at larger linear distances of fragments that interact in mouse primary erythroid cells than in mouse brain cells (Figure 6). This effect can be explained by the fact that the *β-globin* locus is active in mouse primary erythroid cells. Furthermore, we observe that the boundaries of the topological domain, which contains the *β-globin* locus, are easily observed in mouse erythroid cells (Figure 3A). That is prominent when depicting only the Ctcf interacting fragments (Figure 5C *vs.* Figure 5D). Furthermore, the number of interactions within that topological domain, appear higher in the erythroid cells comparing to fetal brain cells (Figure 3A *vs.* Figure 3B, Figure 6A, B). We hypothesize that this is due to the fact that the *β* -globin locus is active with open chromatin in mouse primary erythroid cells. Hence, the chromatin has a different conformation by enabling the interaction between many different elements necessary for the regulation of the gene [34]. However, in mouse fetal brain cells, where *β-globin* locus is not active, that is not necessary and there are no important elements that need to spatially be in close proximity.

**Figure 7:**
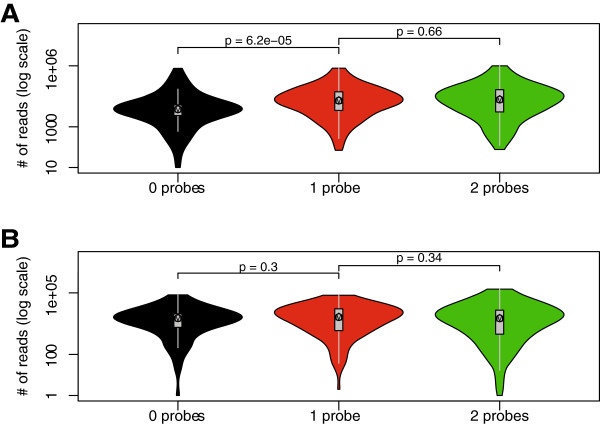
**Comparison of capture efficiencies.** The efficiency with which each fragment of the selected area is captured was derived from counting all of the reads for any particular fragment, that is, all its interactions, its self-ligation, and non-cleaved material and plotting these against the presence of two, one, or no oligonucleotides (probes) in the fragment **(A)**. This shows that the presence of one or two oligonucleotides does not make a difference in the capture as would be expected under conditions where the oligonucleotides are in saturation. When no oligonucleotides are present for a particular fragment, the number of reads will be lower, because the reads due to self-ligation cannot be captured. When the reads are corrected for the self-ligation and non-cleaved fragments this difference largely disappears **(B)**. *P* values were calculated using the Mann–Whitney *U* test.

The method may be improved by bringing the cost further down. For example each of the *β-globin* locus experiments was carried out by using one sequencing lane on an Illumina HiSeq machine for each different biological sample (mouse primary erythoid cells and mouse fetal brain cells). That yielded after comprehensive data analysis and 271,177 and 557,763 paired-reads within the limits of the region of interest excluding self-ligations and uncut fragments for both fetal brain and liver (see Methods). These reads represented 2,369 and 4,057 distinct interactions with 114 and 137 reads per interaction on average for fetal brain and liver, respectively (Table 2). The read frequency of the highest 20% of the interactions is from 11,858 to 202 in fetal liver and from 29,637 to 188 (the top 30% is from 11,858 to 123 and 29,637 to 120 for fetal liver and fetal brain, respectively). The bottom 20% account for four reads in both tissues (while 30% account for nine and 13 for fetal liver and fetal brain, respectively). The question then becomes whether one could do more samples per lane (that is, a reduction in cost per sample) which would result in fewer reads per interaction point. The decision on this depends to some extent on the research question asked. Analysis of functional interactions and/or the ‘rough’ overall structure of a locus, can be achieved by using a range between 1/2 and 1/13 of a sequencing lane which will dramatically lower the costs without losing much information.We also considered using mechanical shearing instead of a secondary restriction enzyme. The advantage of the secondary restriction enzyme over mechanically shearing is that it is very reproducible and provides a better repair step of the ends and hence ligation of the adapters. The possible disadvantage of the second cleavage would seem to be a loss of fragment, because a number of fragments would be represented by one or no oligonucleotide. However when the oligonucleotides are used in excess, as in T2C, there is virtually no statistically significant difference in detecting the reads of fragments represented by two, one, or no oligonucleotides (Figure 7). Mechanically shearing would have the advantage that the chance of capturing a fragment is improved, because some of the secondary restriction sites are too close to the primary restriction sites. However the disadvantages are that mechanically shearing is random, which will have the same possible loss addressed above, but more importantly mechanical shearing is difficult to standardize between different laboratories. Using two different sets of oligonucleotides in combination with two different restriction enzymes for the first or second cleavage would give the most advantage because fewer fragments would be lost and the overall resolution and coverage would be further improved.The ‘quantification’ could be further improved by spiking the samples with control cells preferably from another species, to allow easy recognition of the spike when mapping the sequences back to the genome during the analysis of the ligated fragments. This would also require the addition of a spike specific set of capturing oligonucleotides. Spiking the sample with a DNA sample with a different address sequence at the amplification and sequencing stage of the procedure would also be an improvement, although it would be less quantitative than the spiking with cells at the start of the procedure. The normalization of the signals using the capture efficiency of each of the fragments (Figure 7) also increases the ‘quantification’, although it should be noted these are all relative numbers rather than a real quantification because a number of parameters cannot be controlled or assessed properly.

Because T2C is focused on particular regions of interest, it would be easy to design a set of oligonucleotides for a number of loci that are known to be associated with a particular disease and design a diagnostic kit on that basis that could handle many samples at the same time. Since SNPs are often linked to diseases, dedicated oligonucleotides for them can be designed in order to assess their effect in long range interactions and the regulation of the gene transcription. For non-clinical research purposes the size of the region used in our experiments is sufficient (more than 2 Mb) to extract safe conclusions about the local chromatin interactome and the compartmentalization of the genome.

## Conclusions

We conclude that T2C can be used as an affordable, cost-effective, diagnostic tool with single restriction fragment resolution to explore the local spatial organization of the genome and chromatin interactions without requiring laborious procedures or massive sequencing efforts.

## Methods

### Oligonucleotide design

A microarray for the β-globin locus was designed containing unique oligonucleotides and physically as close as possible to the HindIII restriction sites spanning 2.1 Mb around the gene (chr7: 109876329-111966581, mm9). For the *H19/IGF2* locus unique oligonucleotides were designed close to BglII restriction sites (chr11:1100646-3173091, hg18) spanning an area of 2.1 Mb (Table 2).

The oligonucleotides were designed with the following criteria, they should be: (1) as close as possible to the first restriction site; (2) a unique DNA sequence within the area of interest and preferably in the entire genome; (3) similar melting temperatures, but with different base composition and the length; (4) oligonucleotides which exceed the second restriction site due to very small end fragments, were trimmed keeping in mind to stay close to the same melting temperature.

A custom-made NimbleGen Sequence Capture 2.1 M capture array is produced separately for the *H19/IGF2* locus and for the *β-globin* locus containing for each one the oligonucleotides which satisfy the aforementioned criteria. The oligonucleotides, 525 for the *H19/IGF2* locus and 800 for the *β-globin* locus, were replicated proportionally and equally up to 2.1 M in total for each design, that is, for the *β-globin* locus each of the 800 oligonucleotides was spotted in 2,625 spots.

### Chromatin isolation and library preparation

Nuclei from approximately 10^7^ mouse primary erythroid cells from mouse fetal liver E12.5, mouse fetal brain cells E12.5, and a human breast endothelial cell line (HB2) were isolated, cross-linked (in 2% formaldehyde at room temperature) quenched with 1 M glycine and were re-suspended in lysis buffer (10 mM Tris–HCl (pH 8.0), 10 mM NaCl, 0.2% (vol/vol) NP-40 and 1× protease inhibitor solution). The chromatin was digested with a 6-cutter (400 units of *Hin*dIII for mouse cells and *Bgl*II for the HB2 cells) and ligated using 100 units of T4 DNA ligase (Promega) under conditions favoring intramolecular ligation events. After reversing the cross-link at 65°C overnight, 50 μg of the resulting DNA chromatin library were digested with a frequent 4-cutter (*Dpn*II or *Nla*III for the mouse cells, *Nla*III for the HB2 cells, at a DNA concentration of 100 ng/μL, using 1 unit of enzyme per μg of DNA). All these steps were performed according to the initial steps of 3C-seq protocol, as described previously [23].

The final library is prepared for analysis on the Illumina Cluster Station and HiSeq 2000 Sequencer according to the Illumina TruSeq DNA protocol with modifications (http://www.illumina.com). In short, the digested library is purified using AMPure XP beads (Beckman Coulter), end-repaired, and cleaned using AMPure XP beads. The now blunt-ended fragments were A-tailed using the Klenow exo enzyme in the presence of ATP and purified again using AMPure XP beads. Then indexed adapters provided by Illumina were ligated to the A-tailed DNA fragments with subsequent purification using AMPure XP beads.

### Array capturing

The resulting adapter-modified DNA library (300 to 500 ng) was hybridized in 35 μL for 64 h at 42°C on a custom-made NimbleGen Sequence Capture 2.1 M capture array according to NimbleGen Sequence Capture array protocol (http://www.nimblegen.com/seqcapez) on the NimbleGen Hybridization System. The captured DNA fragments are eluted from the capture array and purified using MinElute columns (Qiagen). The yield for a positive region (a fragment inside the region of interest) and a negative region (a fragment outside the region of interest) differ by >30-fold on average. The captured DNA fragments are amplified by 12 PCR cycles. PCR products are purified using AMPure XP beads and eluted in 30 μL of re-suspension buffer. One microliter is loaded on an Agilent Technologies 2100 Bioanalyzer using a DNA 1000 assay to determine the library concentration and to check for quality.

### Cluster generation and high throughput sequencing

Cluster generation is performed according to the Illumina Cluster Reagents preparation protocol (http://www.illumina.com). Briefly, 1 μL of a 10 nM TruSeq DNA library stock DNA is denatured with NaOH, diluted to 9-10 pM and hybridized onto the flowcell. The hybridized fragments are sequentially amplified, linearized, and end-blocked according to the Illumina Paired-end Sequencing user guide protocol. After hybridization of the sequencing primer, sequencing by synthesis is performed using the HiSeq 2000 sequencer with a 101 cycle protocol according to manufacturer’s instructions. The sequenced fragments were denatured with NaOH using the HiSeq 2000 and the index-primer was hybridized onto the fragments. The index was sequenced with a seven-cycle protocol. The fragments are denatured with NaOH, sequentially amplified, linearized, and end-blocked. After hybridization of the sequencing primer, sequencing by synthesis of the third read is performed using the HiSeq 2000 sequencer with a 101-cycle protocol.

### Targeted Chromatin Capture data analysis

The generated HiSeq 2000 sequencing reads were trimmed if the reads contained the first enzyme restriction recognition site (*Hin*dIII for the mouse derived reads and *Bgl*II for the human derived reads) For each read with one or more enzyme recognition sites, the DNA sequence after the 3′ end of the first site was removed, that is, after the trimming procedure the trimmed reads contained and ended with a single restriction recognition site. Subsequently, consecutive bases with a quality score lower than 10 were cut off from the ends of all the reads and the reads that contained less than 12 bases were omitted using Trimmomatic [47]. We used the Burrows-Wheeler Alignment tool (BWA, version 0.6.1) to the whole genome NCBI36/hg18 assembly for the human derived reads and to NCBI37/mm9 assembly for the mouse derived reads, using default settings [48]. Aligned reads that localized between two second enzyme recognition sites that did not contain a first enzyme recognition site, that is, all *Nla*III-*Nla*III restriction fragments were removed using BEDtools [49].

In the alignment, paired reads were removed if one of the reads was not uniquely mapped. Furthermore, paired reads that were a result of a self-ligation event, non-digestion/re-ligation event, or a ligation of identical ends were removed from the analysis, since these paired reads introduce a common bias in chromosome conformation capture techniques [50,51]. The alignments were further processed with SAMtools [48] to generate paired-end Binary Alignment/Map (BAM) files. BEDtools [49] was used to remove reads that overlapped more than one restriction fragment. Interaction matrices were generated from the alignments at a resolution of the restriction fragments and at 40 kb resolution (using BEDtools on a 40 kbp binned genome). In addition, the human T2C 40 kb binned data were compared to IMR90 40 kb Hi-C data of the combined replicates [1]. The T2C interaction plots were normalized for capture efficiency of the fragments. For each interaction the number reads of each interaction was normalized through dividing it by the sum of the reads of both fragments involved in the interaction. Similarly, the T2C plots of the 40 kb bins were normalized after all the fragments were divided into 40 kb bins along each chromosome. ChIP-seq and T2C interaction-intersection plots were generated from normalized T2C interaction plots and intersected with fragments that contained a ChIP-seq peak signal of the protein of interest. The statistical software package R (version 3.1.0) was used to generate the interaction plots and to conduct the statistical calculations [52].

### ChIP-seq analysis

Published ChIP-seq datasets [37,38] were obtained and analyzed. MACS [53] was used to identify peaks (fdr ≤0.01, peak height ≥20 overlapping reads) to intersect their positions with the interacting fragments obtained from T2C.

## Competing interests

The authors declare that they have no competing interest.

## Authors’ contributions

PK, KSW, TAK, and FG designed the experiments. PK, JZ, and KSW carried out the experiments. HJGvdW performed the bioinformatics analysis. NK and RWWB conducted the initial steps of bioinformatics analysis. CEMK and WFJvI carried out the Illumina sequencing. PK, HJGvdW and FG wrote the manuscript. All authors read and approved the final manuscript.
